# Cyclophilin Inhibitors Remodel the Endoplasmic Reticulum of HCV-Infected Cells in a Unique Pattern Rendering Cells Impervious to a Reinfection

**DOI:** 10.1371/journal.pone.0159511

**Published:** 2016-07-21

**Authors:** Udayan Chatterji, Michael Bobardt, Lana Schaffer, Malcolm Wood, Philippe A. Gallay

**Affiliations:** 1 Department of Immunology & Microbial Science The Scripps Research Institute, La Jolla, California, 92037, United States of America; 2 DNA Array Core Facility, The Scripps Rese6arch Institute, La Jolla, California, 92037, United States of America; Inserm, U1052, UMR 5286, FRANCE

## Abstract

The mechanisms of action by which cyclophilin inhibitors (CypI) interfere with the HCV life cycle remain poorly understood. We reported that CypI and NS5A inhibitors (NS5Ai), but not other classes of anti-HCV agents, prevent assembly of double membrane vesicles (DMVs), which protect replication complexes. We demonstrated that both NS5A and the isomerase cyclophilin A (CypA) are required for DMV formation. Here, we examined whether CypI mediate an additional antiviral effect that could further explain the high efficacy of CypI. We identified a unique action of CypI. CypI remodel the organization of the endoplasmic reticulum (ER) of HCV-infected cells, but not of uninfected cells. This effect is specific since it was not observed for other classes of anti-HCV agents including NS5Ai, and has no effect on the viability of CypI-treated cells. Since ER serves as platform for the establishment of HCV replication complexes, we asked whether the ER reorganization by CypI would prevent cells from being newly infected. Remarkably, CypI-treated HCV-pre-infected cells remain totally impervious to a reinfection, suggesting that the CypI-mediated ER reorganization prevents a reinfection. This block is not due to residual CypI since CypI-resistant HCV variants also fail to infect these cells. The ER reorganization by CypI is rapid and reversible. This study provides the first evidence that CypI trigger a unique ER reorganization of infected cells, rendering cells transiently impervious to a reinfection. This study further suggests that the HCV-induced ER rearrangement represents a key target for the development of new therapies.

## Introduction

More than 200 million people are affected by chronic hepatitis C, which is a leading cause of acute and chronic liver diseases, and approximately 4 million new HCV infections occur every year [1–2]. Two-thirds of liver cancer and transplant cases in the developed world are caused by hepatitis C [3]. Fortunately, several direct-acting antiviral (DAAs) such as NS3 (NS3i), NS5A (NS5Ai) and NS5B (NS5Bi) inhibitors have been FDA-approved and have shown high efficacy in patients, but the cost of these IFN-free DAA regimens is significantly expensive [4]. One option to decrease the cost of these DAA treatments is to reduce the time of drug administration, while still providing efficacy. However, shortening IFN-free treatments did not result in adequate efficacy in naïve cirrhotic patients, treatment experienced non-cirrhotics or genotype-3 (GT3)-infected patients [5–6]. Because current IFN-free DAA treatments mainly entail identical classes of inhibitors—NS3i, NS5Ai and NS5Bi—it is expected that their costs will be elevated at least for a few years and will offer comparable degrees of efficacy. Furthermore, the emergence of drug resistance and side effects after IFN-free DAA treatments will begin to be detected [7]. Incorporating drugs with distinct mechanisms of action (MoA) into IFN-free DAA regimens could offer an opportunity for reducing the time of DAA treatments and prevent the possibility of the development of drug resistance.

Host-targeting antivirals (HTAs) provide very distinct MoA than DAAs since they target host components rather than viral proteins. Cyclophilin inhibitors (CypI) represent the most advanced HTAs in the treatment of HCV-infected patients. The CypI, alisporivir (ALV), provided high efficacy as HTA treatment with or without IFN in phase II and III studies [8–10]. IFN-free ALV treatment is highly effective in GT2 and 3 patients [8]. This is significant since NS3i, NS5Ai and NS5Bi inhibitors have performed less efficiently in GT3 than other GTs [11–12]. Therefore, CypI represent an attractive addition to current IFN-free DAA regimens, at least for GT3 patients.

However, the MoA of CypI remain obscure. We and others demonstrated that CypI target the host protein cyclophilin A (CypA) and that CypA via its isomerase and/or ligand binding activity is absolutely necessary for HCV replication [13–16]. We showed that by binding to the isomerase pocket of CypA, CypI inhibit interactions between CypA and the HCV NS5A protein derived from different GTs [17–21]. Since CypI mediate a pangenotypic antiviral activity (at least for GT1 to 4), our findings suggest that CypA-binding to NS5A is a prerequisite for HCV replication [22–24]. Although the Lippens lab demonstrated by nuclear magnetic resonance (NMR) that CypA isomerizes peptidyl-prolyl bonds in the domain II of NS5A [18], we still do not know whether this folding is important for HCV replication. Since the hydrophobic pocket contains both the isomerase and ligand binding activities of CypA, one cannot determine which of these two actions are required for HCV replication. We and others showed that CypI exhibit a high barrier to resistance both *in vitro* and *in vivo*, and that multiple NS5A mutations are required for CypI resistance (~2-4-fold resistance) [25–27]. This is important since recent studies showed that resistance-associated variants persist for several years post-treatment in patients exposed to NS5Ai or NS5Bi who fail to achieve an sustained viral response (SVR) [7–8, 11–12], possibly impairing their chance for cure on retreatment with existing DAA combinations. Because of their high barrier to resistance, CypI may be extremely useful in combination with NS5Bi as a rescue therapy for patients who relapse with DAA resistance-associated variants. We also demonstrated that the resistance mutations, which emerge *in vitro* under CypI selection, do not render NS5A-CypA interactions impervious to CypI disruption [17]. However, they allow HCV to replicate in CypA-knockdown (KD) cells [25, 28], suggesting that mutations in the domain II of NS5A render HCV partially CypA-independent. More recently, we demonstrated that a combination of CypI (ALV) and NS5Ai (daclatasvir) provides an additive effect on GT1 and 4 and synergistic effect on GT2 to 3 [29]. The idea of using two classes of drugs acting directly (NS5Ai) and indirectly (CypI) on NS5A via distinct domains (domain I and II, respectively) is appealing. CypI due to their pangenotypic potential and high barrier to resistance could represent cornerstone drug partners for DAAs as IFN-free regimens. Nevertheless, their MoA should be elucidated and the role of CypA in the HCV life cycle should be fully understood.

The Bartenschlager lab [30], and more recently, our lab [31–32], demonstrated that CypI such as cyclosporine D (CsD), ALV and CPI-431-32 inhibit the formation of double membrane vesicles (DMVs), which are thought to function as membranous compartments where HCV replication occurs efficiently and safely, protected from cellular RNA sensors and degradation factors [33–34]. The Bartenschlager lab as well as others including ours demonstrated that NS5Ai, like CypI, also inhibit the formation of DMVs [31, 35–36]. Importantly, we showed that no other classes of anti-HCV agents—NS3i, NS5Bi, mir-122 (mir-122i) or phosphatidylinositol-4 kinase IIIα inhibitors (PI4KIIIαi)—inhibit the formation of DMVs formed by HCV [31]. During the course of this study, we discovered that CypI mediate a unique effect on the ER organization of HCV-infected cells. Specifically, we found that CypI entirely restructured the membranous web (MV) structure that was specifically created by HCV to facilitate its replication. This effect is specific since it was not observed for other classes of anti-HCV agents including NS5Ai. Remarkably, CypI-treated HCV-pre-infected cells remained totally impervious to a second infection, suggesting that the CypI-mediated ER reorganization prevents a second HCV infection. This block is not due to residual CypI since CypI-resistant HCV variants also fail to infect these cells. Our study provides the first evidence that CypI elicit a unique ER re-arrangement of infected cells, rendering cells resistant to a reinfection.

## Materials and Methods

### Anti-HCV agents

The HCV NS5Ai daclatasvir (Daklinza) (Bristol Myers Squibb) and ledipasvir (Gilead), the NS5Bi sofosbuvir (Sovaldi) (Gilead) and mericitabine (Roche), the NS3i boceprevir (Victrelis) (Merck) and telaprevir (Incivek) (Vertex) were all obtained from MedChemexpress (Princeton, NJ 08540, USA). The CypI alisporivir (ALV) was provided by Novartis Pharma, Basel, Switzerland or obtained from Acme Bioscience, CPI-431-32 from Ciclophilin Pharmaceuticals, San Diego, USA, and cyclosporine A (CsA) from Sigma-Aldrich, St. Louis, USA.

### Plasmids and cells

The full genomic luciferase reporter replicon Luc-Neo-JFH-1 was created as we described previously [37] while JFH-1-FL (full-length w/o reporter gene) and JFH1-sgR-Neo (sub-genomic) were generously obtained from Drs. Wakita and Chisari [38]. The CypI-partially resistant JFH-1 D316E/Y317N plasmid [28] was created as we described previously [29]. The GT1b subgenomic *firefly* luciferase reporter replicon pFK-I389/NS3-3’ [39] was generously provided by Dr. Bartenschlager.

### EM analyses

JFH-1-infected Huh7.5.1 cells derived from a stable cell line, which we created previously [37] were plated in (350,000/ Falcon 35 mm dish) in triplicate. Twenty-four hours post-plating, drugs were added to cells for 24 h and supernatant replaced with fresh medium. Cells were then fixed with 2.5% glutaraldehyde in 0.1 M cacodylate buffer, washed quickly, post-fixed in cacodylate buffered 1% osmium tetroxide with 1% potassium ferricyanide. Cells after an additional buffer wash were dehydrated in ethanol cycles, shifted in 2-hydroxypropyl methacrylate and embedded in LX112 (Ladd Research, Williston, VT). Parts of cell-containing embedded resin were glued to a blank block face and cut as 60 nm thin sections, which were then mounted on copper slot grids coated with parlodion and stained with uranyl acetate and lead citrate for analysis on a Philips CM100 electron microscope (FEI, Hillsbrough OR) at 80 kv. Images were collected using a Megaview III ccd camera (Olympus Soft Imaging Solutions GmbH, Münster, Germany). Thin sections were cut in the direction parallel to the substrate, and the slice producing the largest nuclear diameter was analyzed, since this plane was generally found to contain the largest number of DMVs. Electron micrographs (between 20 and 100) covering the entire cross-section of the cell were recorded, and to facilitate counting, these were digitally merged to produce a single image representing a 60-nm-thick plane through the center of the infected cell. Merged images were analyzed using ImageJ software (http://rsb.info.nih.gov/ij/).

### First and Second Infections

Luc-Neo-JFH-1 infectious particles (5 x 10^3^ TCID_50_) were collected from the Luc-Neo-JFH-1 stable cell line and used to infect naïve or JFH-1-infected Huh7.5.1 cells at a multiplicity of infection (MOI) of 1. Luciferase activity in lysates of infected cells was quantified 48 h post-2^nd^ infection. Luc-Neo-JFH-1 D316E/Y317N particles were derived from electroporated Huh7.5.1 cells as described previously [37].

### Viral Entry Assay

JFH-1-infected cells, pretreated with ALV for 24 h to induce ER distension, were exposed to JFH-1 (5 x 10^3^ TCID_50_) for 4 h in the presence of DMSO or ALV. Cells were then washed three times and lysed. Protein concentration levels in cell lysates were standardized using the BCA Protein Assay Kit (Biovision Inc.). Amounts of internalized virus were then quantified by HCV core ELISA according to the manufacturer’s instructions (Ortho HCV antigen ELISA kit; Ortho Clinical Diagnostics distributed by Waco Chemicals). Cells were trypsinized 15 min before adding the virus to remove cell surface receptors and inhibit viral entry as control.

### CypA-NS5A Complex Pulldown Assay

JFH-1-infected cells were treated with DMSO or ALV for 24 h. Cell lysates were pre-cleared with agarose beads and incubated for 6 h at 4°C on a wheel with anti-CypA IgG covalently linked to agarose beads. Beads were washed and eluted material analyzed by SDS-PAGE. NS5A and CypA were detected by Western blotting using anti-NS5A (ViroStat) and anti-CypA antibodies (The Scripps Research Institute (TSRI), Antibody Core Facility).

## Results

### CypI, but not other anti-HCV agents, remodel the organization of the ER of HCV-infected cells

**Fig 1:**
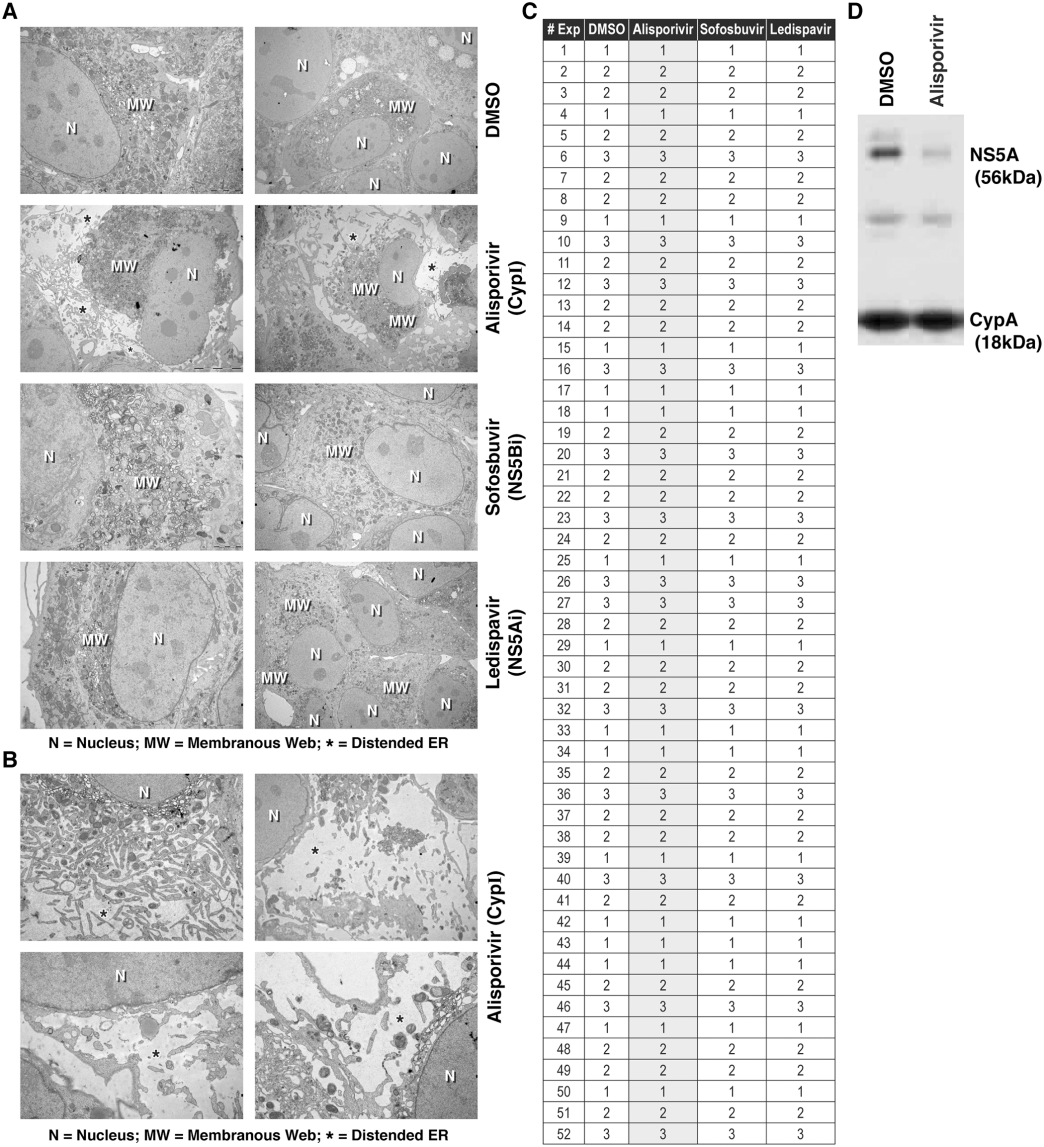
The CypI alisporivir, but not other anti-HCV agents such as the NS5Bi sofosbuvir and the NS5Ai ledispavir, remodel the organization of the ER of HCV-infected cells. A. JFH-1-infected Huh7.5.1 cells were treated with DMSO control or selected drugs—ALV 1 μM, sofosbuvir 5 μM and ledispavir 200 nM—for 24 h and analyzed by EM. N = Nucleus; MW = membranous web; and * = distended ER. B. Same as A except that ER distension is shown at higher magnification. Images are representative of more than 20 independent experiments. C. Number of cells containing distended ER per 100 cells analyzed in 52 independent experiments. JFH-1-infected cells were treated the same as A. Statistic analyses are presented. Paired 2-sample t-tests were conducted to compare the effects of ALV, sofosbuvir, or ledispavir to the DMSO control. D. JFH-1-infected cells were treated with DMSO or ALV for 24 h. Cell lysates were incubated with anti-CypA IgG covalently linked to beads. Beads were washed and eluted material analyzed by SDS-PAGE using anti-NS5A and anti-CypA IgG.

A common feature of all positive-strand RNA viruses is the remodeling of intracellular membranes creating mini-organelles or replication factories where RNA amplification takes place [33, 40–42]. Formation of such sites facilitates coordination of the steps of the replication cycle, and shield HCV dsRNA, from innate sensors. Membrane rearrangements with a membranous web (MW)-like appearance were detected in cells over-expressing the viral polyprotein or NS4B [33, 40, 43–44]. The MW, derived from the ER, is a cytoplasmic accumulation of heterogeneous membranous vesicles embedded into an amorphous matrix. The remodeling of the ER by HCV represents a critical step in its viral replication cycle [40]. Since CypI block an early step of HCV replication, we examined the effect of CypI on the structure of organelles critical for HCV replication. HCV JFH-1-infected Huh7.5.1 cells were exposed to control DMSO or anti-HCV agents including the CypI ALV and the two components of Harvoni used to treat HCV patients—the NS5Bi sofosbuvir and the NS5Ai ledispavir. After 24 h, cells were analyzed by EM as we described previously. We chose 24 h as period of drug treatment since this drug duration suffices to block HCV replication. We confirmed that in the absence of drug (DMSO control), HCV mediates a tremendous ER re-organization and creates the MW close to the nucleus where the virus is presumed to replicate its RNA efficiently (Fig 1A). In contrast to the NS5Bi, sofosbuvir, and the NS5Ai, ledispavir, that do not significantly influence the ER structure of HCV-infected cells, we found that the CypI, ALV, markedly alters the structure of the ER of infected cells (Fig 1A). Although ALV did not completely eliminate the MW, ALV induced a profound remodeling of the ER such as a distension of the membranous structure of HCV-infected cells (Fig 1A, signaled by “asterisks”). Fig 1B shows how ER regions can undergo massive membranous reorganization. As expected, ALV, sofosbuvir and ledispavir do not affect the “normal” structure of the ER of non-infected cells (data not shown).

Due to space consideration, only two pictures are presented per treatment. However, it is critical to emphasize that each selected picture is representative of the phenotypes that we observed from more than 50 independent experiments. Hundreds of cells were analyzed in designated areas for each electron microscope grid, and the same grids were analyzed by two scientists independently. Fig 1C displays the counting (duplicate) of cells containing distended endoplasmic reticulum per 100 cells analyzed in 52 independent experiments. Importantly, the ER distension induced by the 24 h CypI treatment in HCV-infected cells is statistically significant (Fig 1C). Paired 2-sample t-tests were conducted to compare the effects of ALV, sofosbuvir, or ledispavir to the DMSO control. There was a significant difference in the scores found for ALV (M = 64.8, SD = 10.7) and DMSO (M = 1.942, SD = .75) treatments; t(T< = t) = 2.0, p = 2.7E-41. There was no significant difference in the scores found for sofosbuvir (M = 1.961, SD = .684) treatments; t(T< = t) = 2.007, p = .888. There was no significant difference in the scores found for ledispavir (M = 1.942, SD = .725) treatments; t(T< = t) = 2.007, p = 1. The percentages of cells with distended ER are the following: 1.9% for DMSO, 64.8% for ALV, 2.0% for sofosbuvir and 1.9% for ledispavir.

Since we previously showed that CypI prevent the contact between recombinant CypA and NS5A proteins [17], in the context of HCV-infected cells with CypI-induced distended ER, we asked whether CypA-NS5A interactions are also disrupted. Specifically, JFH-1-infected cells were treated with DMSO or ALV for 24 h. Cells were then washed, trypsinized and lysed. Cell lysates were incubated with anti-CypA IgG covalently linked to agarose beads. Beads were washed and eluted material analyzed by SDS-PAGE. Importantly, we found that ALV, but not DMSO treatment of infected cells, prevents the pulldown of NS5A by CypA (Fig 1D). This result indicates that CypA-NS5A complexes are disrupted in ALV-treated cells displaying a distended ER.

### All CypI possess the ability to profoundly alter the ER structure of HCV-infected cells

**Fig 2:**
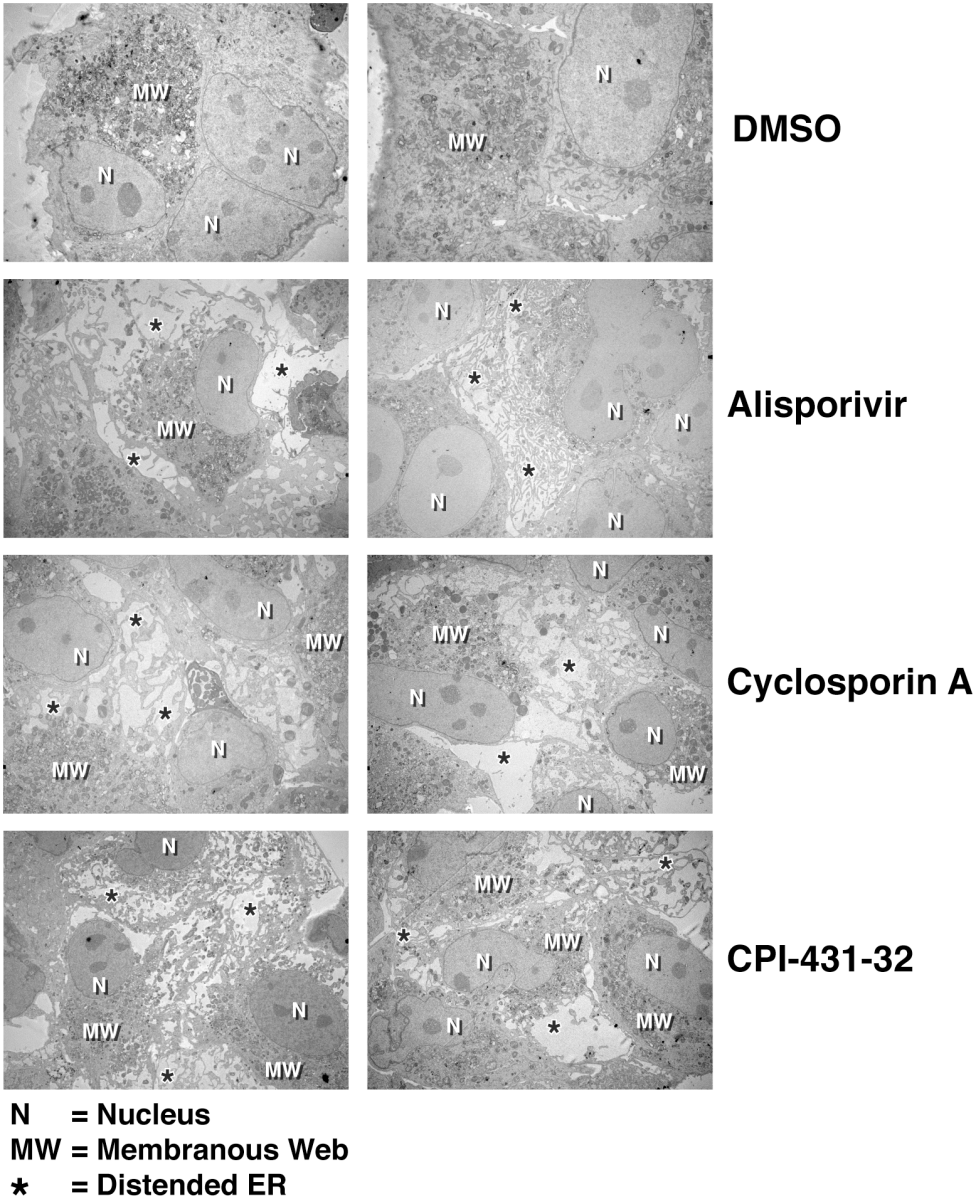
All CypI tested greatly alter the organization of the ER of HCV-infected cells. Same as Fig 1 except that two additional CypI were used—the immunosuppressive CypI CsA and the non-immunosuppressive CypI CPI-431-32. N = Nucleus; MW = membranous web; and * = distended ER. Images are representative of more than 3 independent experiments.

We then asked whether the remodeling of the structure of the ER of infected cells is specific to ALV or is a common feature of CypI. To test this hypothesis, we compared the effect of ALV with that of two other CypI—CsA and CPI-431-32 —which efficiently inhibit HCV replication *in vitro* [32, 45]. In contrast to DMSO, we found that like ALV, both CsA and CPI-431-32 induce a major reorganization of the ER structure of HCV-infected cells (Fig 2).

### Kinetic analysis of the CypI-mediated remodeling of the ER structure of HCV-infected cells

**Fig 3:**
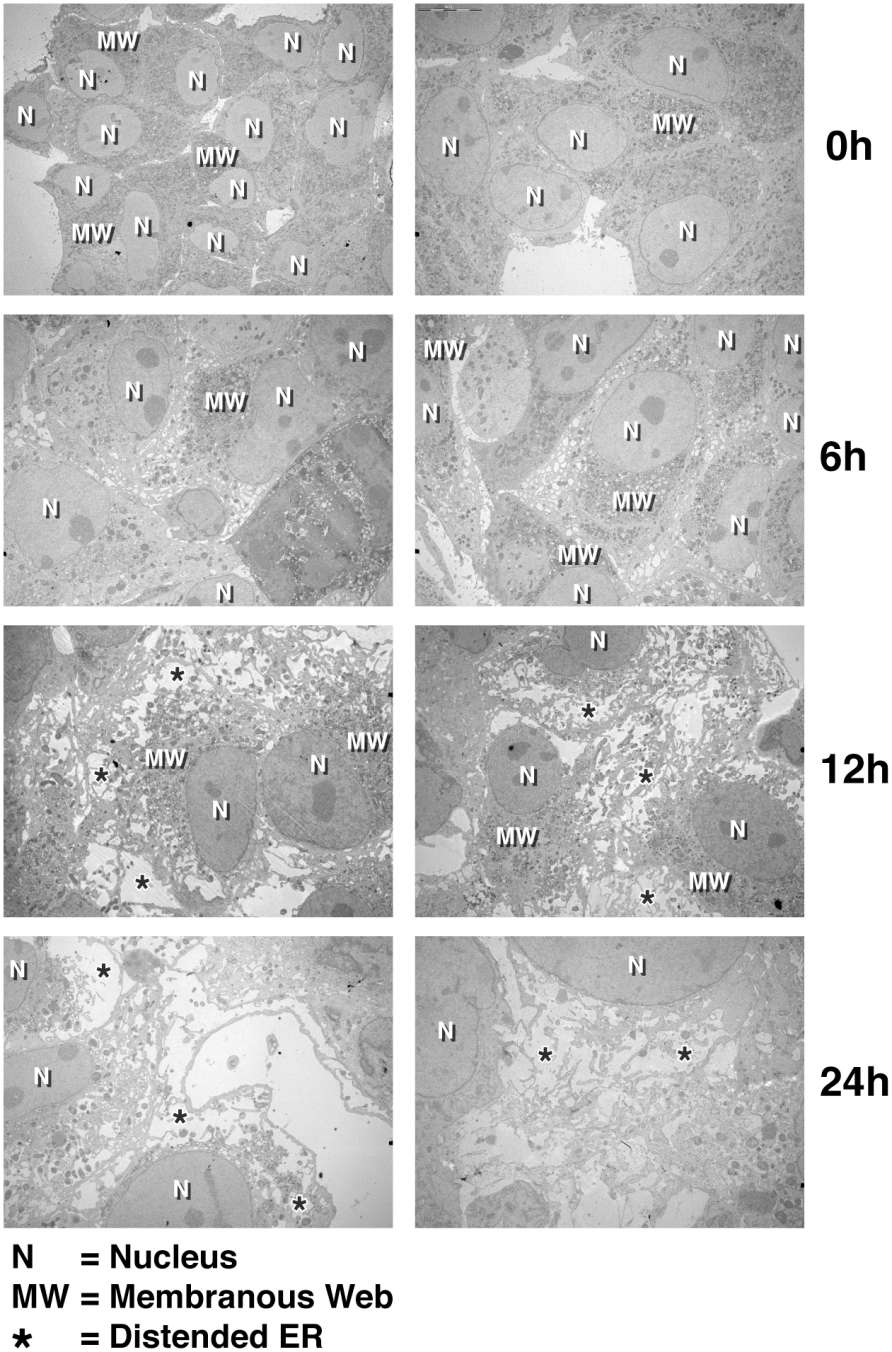
Kinetic analysis of the remodeling of the ER structure of HCV-infected cells by CypI. JFH-1-infected Huh7.5.1 cells were treated with ALV (1 μM) and analyzed by EM at the indicated time points. N = Nucleus; MW = membranous web; and * = distended ER. Images are representative of more than 5 independent experiments.

We then asked how long it takes to CypI to alter the structure organization of the ER of HCV-infected cells. To address this issue, infected cells were exposed to ALV and examined by EM at various time points. Although an ER “vacuolar” phenotype could be observed after 6 h of drug treatment, a more obvious reorganization of the ER structure was observed after 12 h (Fig 3). This indicates that CypI possess the ability to rapidly remodel the structure of the ER in HCV-infected cells.

### The remodeling of the ER structure of HCV-infected cells by CypI is reversible

**Fig 4:**
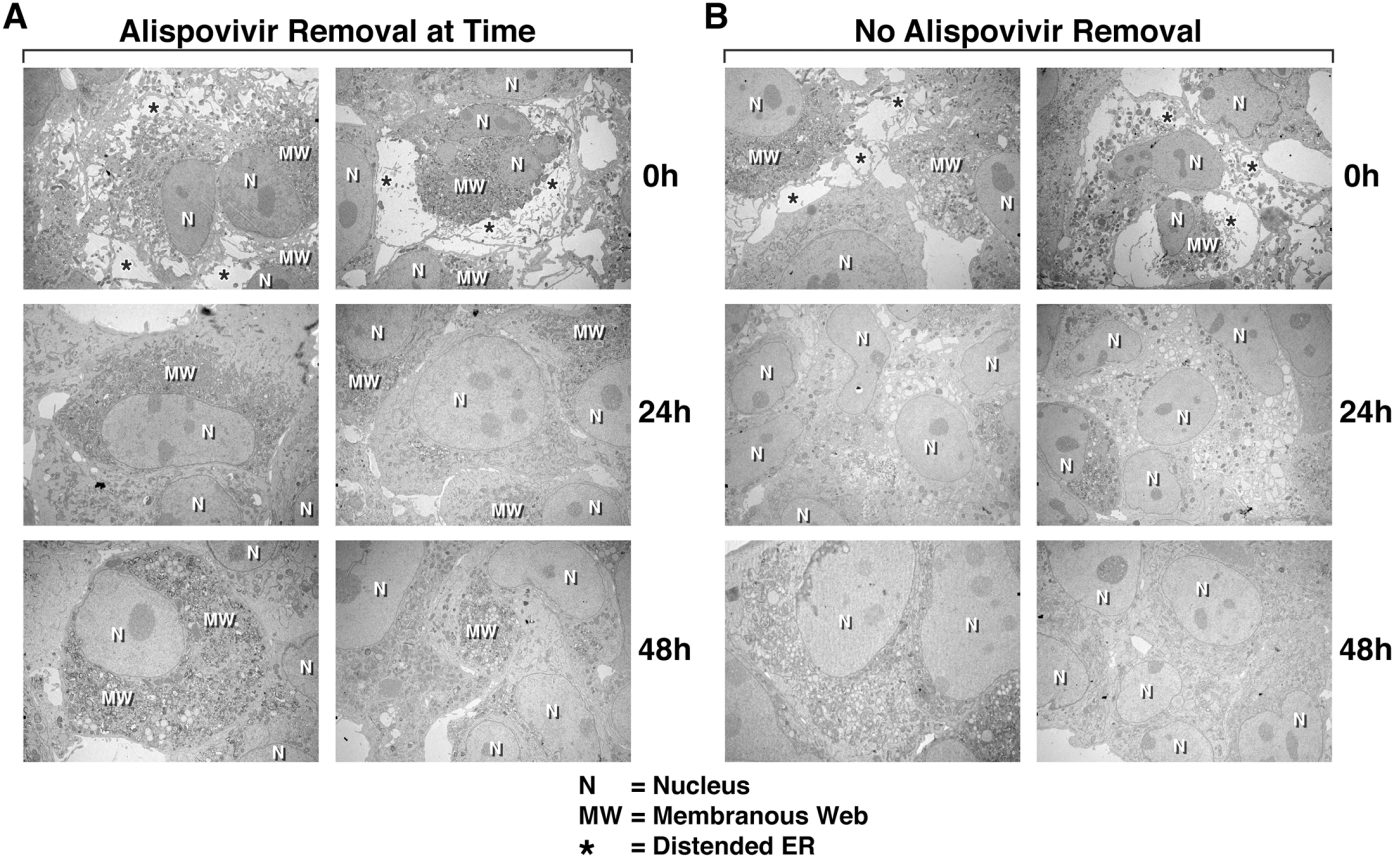
Re-establishment analyses of the MW structure of HCV-infected cells. JFH-1-infected Huh7.5.1 cells were treated with ALV (1 μM) for 24 h, washed to remove the CypI (A) or not (B), and analyzed by EM at the indicated time points. N = Nucleus; MW = membranous web; and * = distended ER. Images are representative of more than 4 independent experiments.

To test whether the CypI effect on MW remodeling is permanent or reversible, HCV-infected cells were treated with ALV for 24 h, extensively washed to remove the drug, and ER structures examined by EM over time. We found that already 24 h after drug removal, the MW structure of infected cells was restored (Fig 4A), supporting the notion that the CypI-mediated ER reorganization is reversible. After diligently counting cells, we calculated the following percentages: 2.2% of cells with distended ER before ALV addition, 64.3% 24 h after ALV addition and 6.3% and 2.5% 24 h and 48 h after ALV removal, respectively. If ALV was not washed away and remained in culture, the ER structure of infected cells was restored after 72 h (time point 48 h), further suggesting a reversible effect of CypI on the intracellular membrane organization. A “vacuolar” pattern could be observed after 48 h of drug treatment (time point 24 h), indicating that the restoration of the ER organization is a continuing process.

### CypI remodel the ER of HCV-infected cells in a unique pattern rendering cells impervious to a reinfection

**Fig 5:**
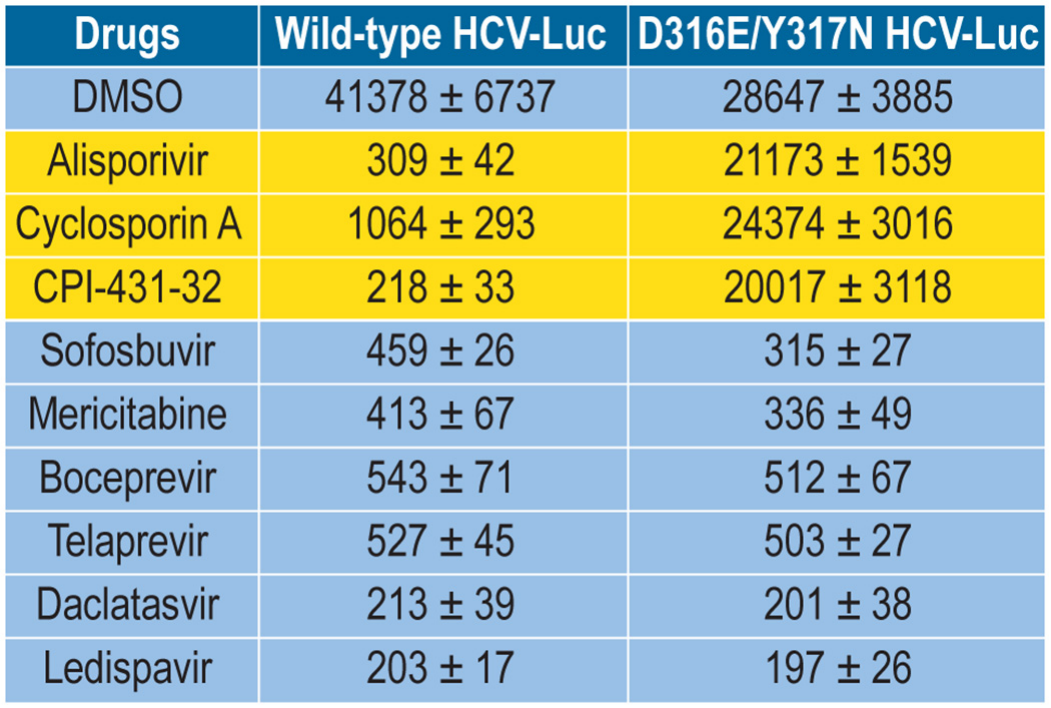
Efficacy of selected anti-HCV agents against wild-type and CypI-resistant HCV. Huh7.5.1 cells were infected with wild-type or CypI-resistant D316E/Y317N JFH-1-Luc (MOI of 1) together with selected anti-HCV agents—ALV (1 μM), CsA A (1 μM), CPI-431-32 (1 μM), sofosbuvir (5 μM), mericitabine (10 μM), boceprevir (5 μM), telaprevir (5 μM), daclatasvir (10 nM) and ledispavir (200 nM) and infectivity scored after 48 h by quantifying luciferase activity in cell lysates. Data (triplicate) are expressed in relative light units (RLU). Data are representative of two independent experiments.

Since we found that CypI remodel the structure of the ER of infected cells in a unique pattern, we asked whether this intracellular membrane reorganization would affect a second HCV infection. Indeed, it is well known that the ER serves as a critical platform for the formation of enzymatically active replication complexes [33–34, 40–42], thus if this membranous platform is perturbed, one can envision that the success of a second infection would be low. To test this hypothesis, we infected cells first with non-reporter infectious particles (JFH-1; i.e. “pre-infected cells”), followed by optional drug treatment, and finally by infection with reporter infectious particles (JFH-1-Luc). Luciferase content in cell lysates was quantified 48 h post-2^nd^ infection. Drug treatments for 24 h between the two infections included CypI (ALV, CsA and CPI-431-32), NS5Bi (sofosbuvir and mericitabine), NS3i (boceprevir and telaprevir) or NS5Ai (daclatasvir and ledispavir), and cells were extensively washed to remove drugs prior to the reporter particle infection. We first verified the efficacy of the anti-HCV agents. Huh7.5.1 cells were exposed to JFH-1-Luc together with the above anti-HCV agents, and infection was quantified 48 h post-virus and drug exposure. We found that all selected anti-HCV agents significantly inhibited HCV infection compared to control DMSO (Fig 5).

**Fig 6:**
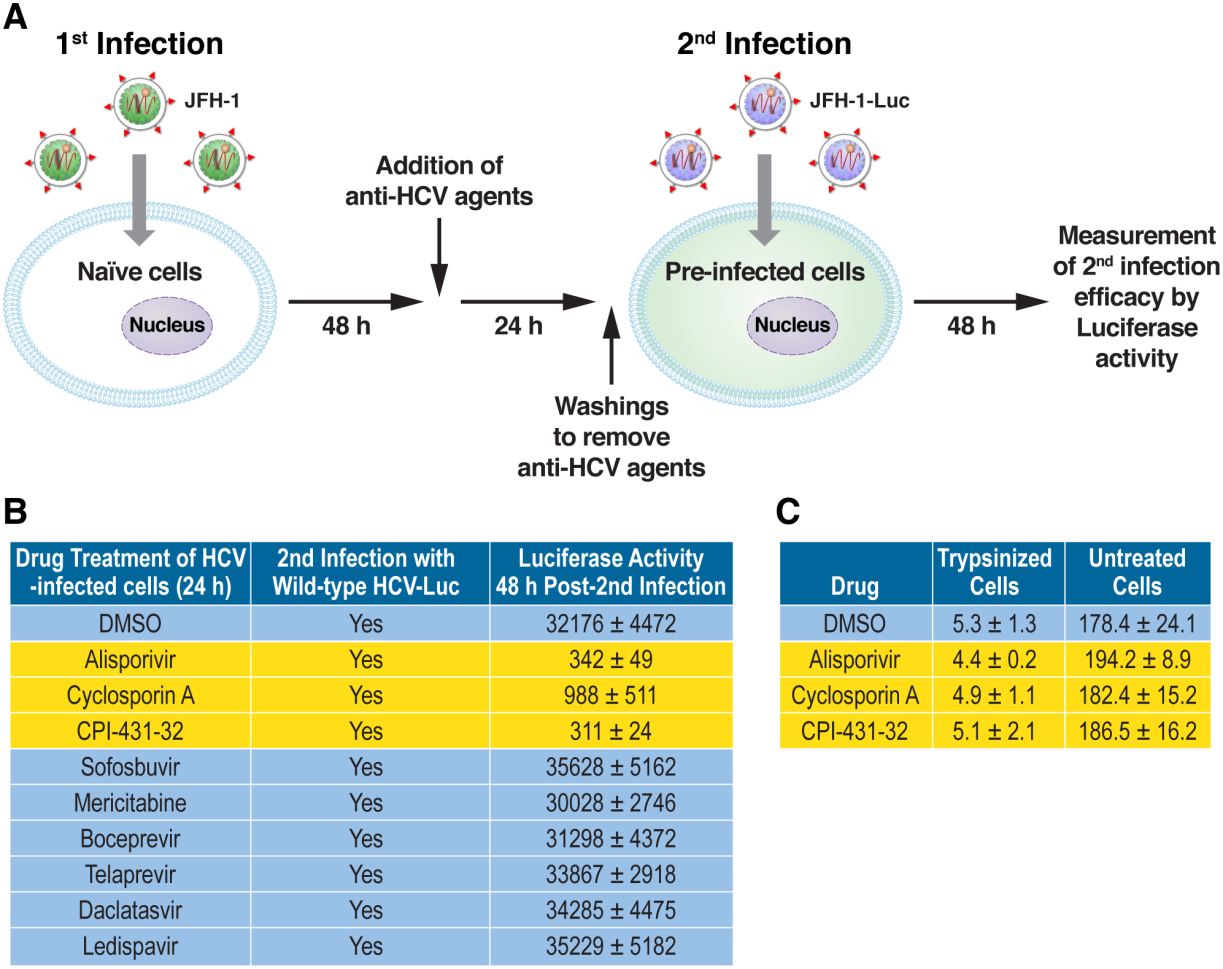
Reinfection analysis of HCV-infected treated with or without selected anti-HCV agents. A. Experimental design for the reinfection assay. B. JFH-1 (no luciferase reporter gene)-infected Huh7.5.1 cells were treated with selected anti-HCV agents—ALV (1 μM), cyclosporine A (1 μM), CPI-431-32 (1 μM), sofosbuvir (5 μM), mericitabine (10 μM), boceprevir (5 μM), telaprevir (5 μM), daclatasvir (10 nM) and ledispavir (200 nM) for 24 h, washed to remove the drugs and re-exposed to JFH-1-Luc (MOI of 1). Reinfection was scored after 48 h by quantifying luciferase activity in cell lysates. Data (triplicate) are expressed in RLU. Data are representative of 4 independent experiments. C. JFH-1-infected cells, pretreated with ALV (1 μM) for 24 h to induce ER distension, were exposed to JFH-1 particles for 4 h in the presence of DMSO or ALV. Cells were then washed three times and lysed. Amounts of internalized virus were quantified by HCV core ELISA. As control, cells were trypsinized 15 min before adding the virus to remove cell surface receptors and inhibit viral entry. Data (triplicate) are expressed as pg of HCV core/mL of cell lysate.

After verifying the efficacy of all drugs, we conducted reinfection assays as described above (Fig 6A). We first found that DMSO-treated pre-infected cells were significantly re-infected with JFH-1-Luc (Fig 6B). Moreover, we found that pre-infected cells, which were treated with NS5Bi, NS3i as well as NS5Ai, and washed, could also be re-infected (Fig 6B). This finding suggests that those drugs were not significantly retained within cells following washout, which permitted a reinfection. In sharp contrast, we found that the efficacy of a reinfection of CypI-treated pre-infected cells was very poor compared that of DMSO- or DAA-treated pre-infected cells (Fig 6B). This finding suggests that the CypI-mediated reorganization of the structure of the MW precluded the initiation of a successful re-infection, perhaps by preventing the formation of enzymatically active replication complexes in the ER.

To exclude the possibility that the inability of HCV to re-infect CypI-treated cells with distended ER is not the result of a blockade of viral entry, we quantified the amounts of viral particles internalized into cells treated with or without ALV. Specifically, HCV-infected cells, pretreated with CypI for 24 h to induce ER distension, were exposed to JFH-1 for 4 h in the presence of DMSO or ALV. Cells were then washed and lysed and amounts of internalized virus were quantified by HCV core ELISA in cell lysates. Similar amounts of core were measured in lysates of DMSO- or ALV-treated cells (Fig 6C), suggesting that the CypI-mediated re-infection block occurs post-entry. As control, cells were trypsinized just before adding the virus to cut off cell surface receptors. As expected, viral entry was greatly diminished by trypsin treatment prior to virus addition (Fig 6C).

**Fig 7:**
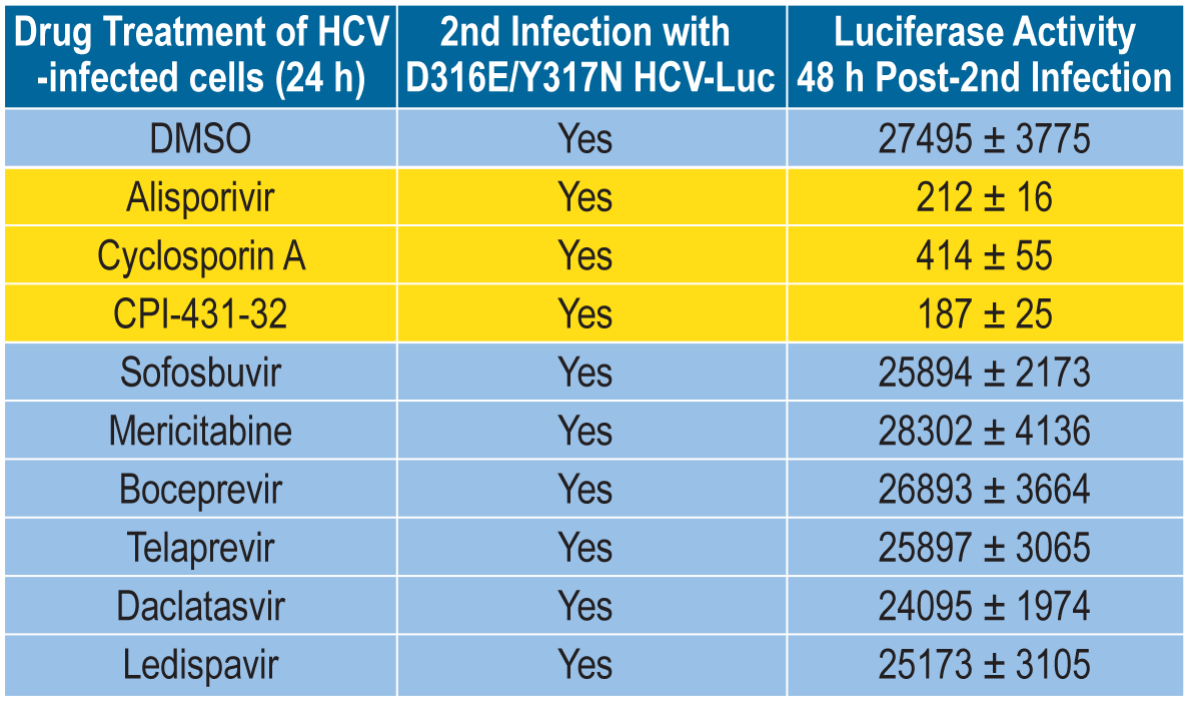
CypI-resistant HCV fails to infect pre-infected hepatocytes treated with CypI, but succeeds to infect pre-infected hepatocytes treated with non-CypI anti-HCV agents. Same as Fig 6 except that the CypI-resistant D316E/Y317N JFH-1-Luc virus was used for the 2^nd^ infection instead of wild-type JFH-1-Luc virus. Data are representative of 2 independent experiments.

To exclude the possibility that residual CypI within pre-infected cells interfere with the reinfection, we took advantage of CypI-resistant HCV variants, which we and others previously identified [18, 21, 25, 28], and asked whether they can infect pre-infected cells, which have been pre-treated with CypI and washed extensively. If some residual CypI remained within pre-infected cells, and if the integrity of the ER platform is preserved enough for the initiation of enzymatically active replication complexes, a reinfection by CypI-resistant variants should be successful. We and others previously identified point mutations in the domain II of NS5A –D320E and Y321N in genotype 1b Con1 and D316E and Y317N in genotype 2a JFH-1 that confer partial resistance to CypI including CsA, ALV and SCY-635 [17–18, 21, 25, 28]. We first verified that D316E/Y317N JFH-1 infects Huh7.5.1 cells even in the presence of CypI. We found that indeed the double substitution D316E/Y317N within NS5A confers significant resistance to CypI, but not to DAAs such as NS5Bi, NS3i and NS5Ai (Fig 5). We next tested the capacity of D316E/Y317N JFH-1 to infect pre-infected cells, which have been treated with a panel of anti-HCV agents including CypI, NS5Bi, NS3i and NS5Ai, and extensively washed prior to the reinfection. Importantly, we found that D316E/Y317N JFH-1 infects efficiently DMSO- as well as DAA-treated pre-infected cells (Fig 7). However, the D316E/Y317N JFH-1 variant was still unable to infect CypI-treated pre-infected cells, suggesting that the inability of wild-type virus to infect CypI-treated pre-infected cells was not the consequence of residual CypI within cells.

## Discussion

Several labs including the Bartenschlager, Roingeard and Tai labs provided evidence that DMVs are membranous compartments created by HCV where the virus initially replicates its genome efficiently and evades the innate response [30, 33–36, 42, 46]. We recently reported that NS5A and CypA, via its ligand binding or peptidyl *cis-trans* isomerase activity, act in concert to create these protective membranous organelles [31]. We also reported that CypI and NS5Ai, but not other classes of anti-HCV agents such as NS3i, NS5Bi, mir-122i or PI4KIIIαi, prevent the formation of DMVs, leading to both a block in viral RNA replication and infection [31]. These findings suggested that preventing the capacity of HCV to shape the structure of the ER into these specific organelles—the DMVs—is deleterious to the virus. As suggested by the Bartenchlager lab, DMVs predominate during the early stages of infection, but they develop into more complexed membrane re-arrangements as infection progresses, probably resulting from a host cell stress response [40].

In the present study, we asked whether CypI exert an action additional to the preclusion of DMV formation that could further explain the high antiviral efficacy of CypI both *in vitro* and *in vivo*. Remarkably, we found that the CypI, ALV, but not DAAs such as the NS5Bi, sofosbuvir, and the NS5Ai, ledispavir, significantly remodels the ER structure of infected cells, but not that of uninfected cells. This effect is not unique to ALV since we showed that other CypI including CsA and CPI-431-32 also mediate this re-organization of the ER. The ER undergoes a dramatic extension, mainly in proximity to the nuclear membrane. How could we explain the specific ER re-organization by CypI? HCV proteins are membrane-associated proteins containing domains allowing their anchoring into the ER membrane [47–48]. Importantly, many of them interact with one another. This ability of HCV components to interconnect likely creates specific ER membrane structures including the MW and DMVs. Importantly, a viral protein of interest for this study—NS5A—forms dimers, binds viral RNA as well as NS5B and NS4B. Since CypA binds and possesses the ability to fold NS5A via its isomerase activity, it can play a critical role in the contact between HCV proteins, and therefore in the modeling of the ER membrane organization. Thus, it is anticipated that CypI, by separating NS5A-CypA complexes, influence the contacts between NS5A and the ER membrane and/or viral components of replication complexes, leading to this particular major ER distension. To the best of our knowledge no ER distension similar to that induced by CypI has been previously observed. The expression of NS5A alone did not induce a distended ER in either DMSO- or CypI-treated cells (data not shown), further suggesting that the presence of all HCV proteins are necessary for the profound reorganization of the ER of cells infected by CypI.

Importantly, the domain I of NS5A is not only responsible to bind the viral RNA, but also for the anchoring of NS5A into the ER membrane [49]. The N-terminus of the domain I of NS5A contains an amphipathic alpha-helical helix critical for ER association [48]. Interestingly, a short peptide called C5A, which encompasses residues 3–20 of the amphipathic α-helical N-terminal membrane anchor domain of NS5A, possesses the ability to block HCV infectivity by disrupting the integrity of the membrane of viral particles [50]. They nicely showed that the amphipathicity of C5A is critical for its virocidal property [50]. In collaboration with several labs including the Chisari lab, we confirmed the ability of C5A to disrupt the organization of viral membrane envelopes including those of HIV, simian immunodeficiency virus (SIV) [51] and herpes simplex virus (HSV) [52]. Together these data strongly suggest that NS5A, via its N-terminal amphipathic α-helical helix possesses the ability to influence the structure of membranes. This is perfectly in accordance with the fact that we and others showed that NS5A alone possesses the ability to create DMVs [30–32, 34–35]. Moreover, the Bartenschlager lab recently demonstrated that the N-terminus of the domain I of NS5A, especially the amphipathic α-helical N-terminus, is vital for the formation of DMVs [53]. Further work is required to determine whether the binding of CypA to NS5A influences the association of NS5A with the ER membrane or with host (i.e., reticulon proteins or other cellular membrane-shaping proteins) or viral components (i.e., NS4B, which is vital to form the MW and which binds NS5A) known to possess the ability modify the ER structure that would lead to the formation of specific membranous structures. Since the new ER re-organization by CypI is not observed in non-infected cells, it is likely that this membranous phenotype triggered by CypI is linked to viral factories. One cannot exclude the possibility that the autophagy machinery or a cellular stress response mediates this distension of the ER structure.

Our finding that CypI, but not other anti-HCV agents, profoundly altered the ER organization of infected cells, strongly suggests that the neutralization of intracellular Cyps, especially CypA, triggers a major rearrangement of intracellular membranes including distension of the ER. We showed that the CypI effect is rapid since a major ER disorganization of infected cells is already detected after 12 h. We also showed the CypI effect is reversible since 24 h after drug removal, the ER organization is fully restored and no severe distension of the ER can be observed. Therefore, the ER re-organization of infected cells by CypI is reversible and harmless for the cells. This is consistent with the observations that CypI such as ALV show little toxicity in *in vitro* [54], in mice [55] or in chronic HCV-infected patients in phase II and III studies [8–10]. Our observation that when the CypI was not washed away and remained in culture, the ER structure of infected cells was restored after several days, further suggested a reversible effect of CypI on the intracellular membrane organization. It also suggested that viral replication and presence of HCV proteins in the ER are preconditions for the CypI-mediated ER distention. Indeed, 48 h of CypI treatment suppresses both viral replication and HCV protein expression in infected cells. This indicates that when viral proteins are no longer present in cells, CypI do not mediate any effect on the ER structure. This is consistent with our observation that CypI have no effect on the ER organization of non-infected cells. A similar CypI-mediated ER re-organization pattern was observed with full-length or subgenomic JFH-1 as well as with subgenomic replicons from genotypes 1b, 3a and 4a (data not shown), suggesting that the ER distension is not dependent of either structural HCV proteins or genotype. This is in accordance with the fact that CypI targets a host protein rather than a viral protein and that CypI are pan-genotypic. Moreover, we showed for the first time that the CypI-mediated reversible ER re-organization renders cells impervious to a second infection. This finding is important because it suggests that CypI exert a dual inhibitory effect on HCV infection: i) they inhibit the first established infection by blocking HCV RNA replication likely by preventing the formation of DMVs, which contain the viral factories; and ii) they prevent a second infection by altering the structure of the ER that HCV normally uses to initiate its replication such as the anchoring of its proteins into the ER membrane and the establishment of enzymatically active replication complexes. This dual antiviral effect may explain the high clinical efficacy of CypI such as ALV in patients infected with any genotypes.

This study provides the first demonstration that CypI, but not other anti-HCV agents including NS3i, NS5Ai and NS5Bi, possess a unique ability that is the alteration of the organization of the ER of infected cells in a such manner that it prevents, at least transiently, a second infection. These new data indicate that CypI block HCV infection and replication by acting at two distinct membranous web biogenesis steps of HCV RNA replication—i) the prevention of the formation of DMVs necessary for shielding viral factories from the innate response; and ii) the reversible destabilization of the ER platform necessary for the establishment of a second infection. Altogether these data further indicate that the architecture and biogenesis of viral factories represent new therapeutic targets of chronic hepatitis C.
